# Effects of apoE Deficiency and Occlusal Disharmony on Amyloid-Beta Production and Spatial Memory in Rats

**DOI:** 10.1371/journal.pone.0074966

**Published:** 2013-09-16

**Authors:** Daisuke Ekuni, Yasumasa Endo, Takaaki Tomofuji, Tetsuji Azuma, Koichiro Irie, Kenta Kasuyama, Manabu Morita

**Affiliations:** Department of Preventive Dentistry, Okayama University Graduate School of Medicine, Dentistry and Pharmaceutical Sciences, Okayama, Japan; Mayo Clinic College of Medicine, United States of America

## Abstract

Amyloid-β (Aβ) plays a causative role in Alzheimer’s disease. Apolipoprotein E (apoE) is involved in Aβ accumulation, whereas occlusal disharmony increases Aβ production in the rat hippocampus. The purpose of the present study was to investigate the effects of apoE deficiency and occlusal disharmony on Aβ production and spatial memory. Wild-type (WT) (n = 12) and apoE-deficient [ApoE(−/−)] (n = 12) rats (Sprague-Dawley; 8 weeks old) were used. These rats were randomly divided into four groups of six rats each: two control (C) groups: WT (C-WT) and ApoE [C-ApoE(−/−)], and two occlusal disharmony (D) groups: WT (D-WT) and ApoE [D-ApoE(−/−)]. The C group received no treatment for 8 weeks. In the D group, the maxillary molar cusps were cut off for 8 weeks. The spatial memory of rats was assessed according to their behavioral performance in a radial arm maze. In both genotypes of rats, significant differences in the reference memory, Aβ42 production, β-secretase expression and plasma corticosterone levels were observed between the C and D groups (*P* < 0.0125). The levels of Aβ42 and glucocorticoid receptor in the C-ApoE(−/−) group were also significantly higher than those in the C-WT group (*P* < 0.0125). However, no significant differences in these parameters were found between the two genotypes with occlusal disharmony. In conclusion, occlusal disharmony induces cognitive dysfunction and Aβ accumulation in the rat hippocampus, and the effects of occlusal disharmony on Aβ accumulation and cognitive dysfunction were larger than those of apoE deficiency.

## Introduction

Alzheimer’s disease (AD), specifically the late-onset form of AD (LOAD), is the most common cause of dementia in individuals older than 60 years of age [1]. Amyloid-β (Aβ) accumulation and its neurotoxicity in the brain play a causative role in AD [2]. Aβ40, Aβ42, and phosphorylated tau reflect the core elements of the disease process in AD [3,4]. Despite intensive laboratory and clinical research over the last three decades, no effective treatment to delay the onset and progression of AD has been established [5].

**Figure 1:**
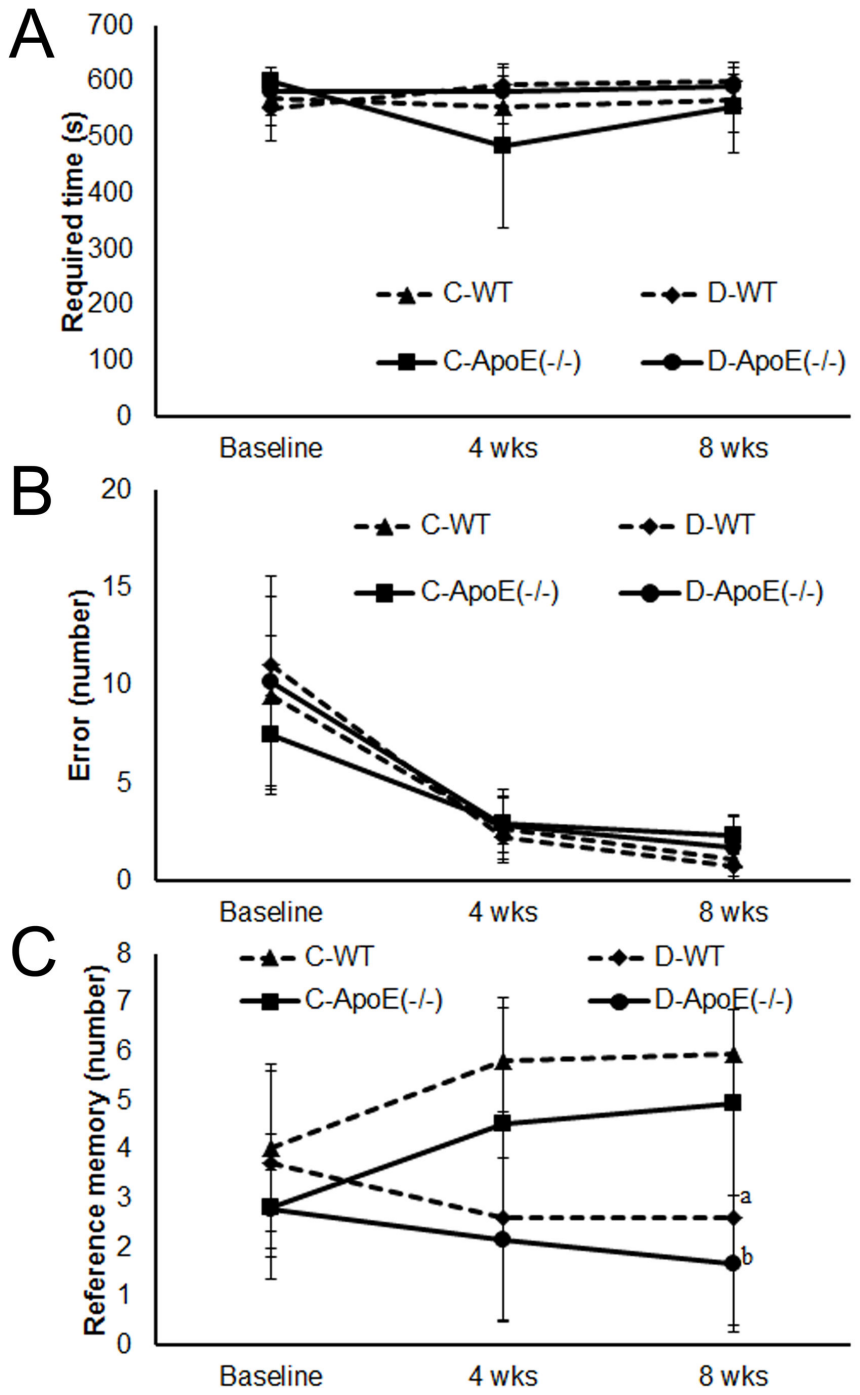
Maze task behavioral performance. The required time (A), error (B), and reference memory (C) are shown in the graphs. For each group, the results are expressed as the mean ± SD (n = 6). C, control; D, occlusal disharmony; WT, wild-type rats; ApoE(−/−), apoE-deficient rats. ^a^ *P* < 0.0125 compared with the C-WT group at 8 weeks (t test with Bonferroni correction). ^b^ *P* < 0.0125 compared with the C-ApoE(−/−) group at 8 weeks (t test with Bonferroni correction).

Although Aβ accumulation is a cause and an effect of AD, an imbalance between Aβ production and clearance is caused by other upstream events in all patients with AD, and most of these other causes are currently unknown [5]. One of the strongest causes is inheritance of one or two ε4 alleles of the apolipoprotein E (apoE) gene [6]. ApoE4 is the major and most prevalent genetic risk factor for AD [6-10]. Furthermore, apoE is present in neuritic plaques and neurofibrillary tangles [11], and apoE4-positive AD patients have higher levels of such plaques and tangles than do corresponding AD patients who do not express this apoE allele [12-16].

When developing a potential pharmacological target of apoE, one must consider that apoE is involved in the clearance of Aβ from the central nervous system across the blood-brain barrier [17,18]. Both apoE-deficient mice and murine apoE-deficiency + human apoE4 mice show impaired clearance of synthetic Aβ injected into the brain parenchyma compared to mice expressing the human apoE3 isoform [19]. Therefore, apoE plays a dual role in Aβ clearance and deposition, which is likely dependent on the concentration of central nervous system Aβ and that of other Aβ binding proteins [20]. Several key questions remain regarding the effect of apoE on Aβ. One unanswered question is whether it is better to increase or decrease human apoE levels (regardless of isoform) to reduce Aβ levels [1].

In addition to apoE, occlusal disharmony may also be a risk factor for AD. A direct relationship between dementia progression and reduced masticatory ability or occlusal force, which may be linked to occlusal disharmony, has been reported in elderly hospitalized patients [21]. In a rat model, psychological stress induced by occlusal disharmony reversibly induces Aβ40 and Aβ42 accumulation in the rat hippocampus through glucocorticoid signaling [22]. These findings suggest that psychological stress following occlusal disharmony may represent a potential risk factor for AD in addition to apoE. Prolonged exposure of older, nondemented, apoE ε4-positive individuals to stress leads to a worse memory decline compared that in apoE ε4-negative individuals [23]. Therefore, the effects of psychological stress following occlusal disharmony on AD progression may differ according to the apoE genotype. However, the combined effects of apoE and occlusal disharmony on the pathways that regulate cellular Aβ levels have not been analyzed.

We hypothesized that apoE and occlusal disharmony modulate Aβ accumulation in the rat hippocampus and affect cognitive function. The purpose of the present study was to investigate the effects of apoE deficiency and occlusal disharmony on Aβ production in the rat hippocampus and spatial memory. In addition, plasma corticosterone concentrations were measured to evaluate the level of psychological stress [22].

## Materials & Methods

### Animals

Twelve male wild-type (WT) and twelve male apoE-deficient [ApoE(−/−)] rats (8 weeks old) (all Sprague-Dawley strain background) were obtained from Sigma Laboratory (St. Louis, MO) for this 8-week study. Animals were housed in an air-conditioned room (23-25°C) with a 12-h light-dark cycle. Animals had free access to a powdered diet (Oriental Yeast Co., Tokyo, Japan) and tap water. The experimental protocol was approved by the Animal Care and Use Committee of Okayama University.

### Experimental design

Both genotypes of rats were randomly divided into two groups of six rats each: a control (C) group, which received no treatment for 8 weeks, and an occlusal disharmony (D) group, in which all maxillary molar crowns were cut off for 8 weeks [22,24]. In the D group, the bilateral maxillary molar crowns were cut off at the gingival margin using a dental turbine [22,24]. Thus, four groups were analyzed in this study: C-WT, D-WT, C-ApoE(−/−), and D-ApoE(−/−). All procedures were performed under general anesthesia by inhalation of 2-4% isoflurane delivered in an O_2_ gas. The animals were sacrificed under general anesthesia after 8 weeks.

### Eight-arm radial maze procedure

The spatial memory of rats was assessed based on their behavioral performance in an eight-arm radial maze (BrainScience Idea Co., Ltd., Osaka, Japan) [25]. At the end of each arm, a 45-mg food pellet was placed on a plate (Bio-Serv, Frenchtown, NJ) [25]. During the training period, groups of two or three rats were released into the central area of the maze and allowed to explore the maze for 3 h per day [25]. At baseline and at 4 and 8 weeks, the maze task was performed once a day for 4 consecutive days [25]. The animals were kept on a restricted diet prior to performing the maze task to motivate the rats to seek the food rewards [26]. Each task began when a rat was placed individually in the center of the maze and terminated when all eight food plates were empty or 10 min had elapsed [26]. The number of correct choices in the initial eight chosen arms was defined as reference memory. The total number of times that the rat entered an arm with an empty plate was defined as the number of errors. To evaluate the rat’s memory and motor performance, the time required to complete the task was defined as the required time. The maze was always set up in the same location. The experiments were conducted at the same time each day. The mean value, which was calculated based on the 4 days of results, was used for evaluation [25].

### Measurement of plasma corticosterone levels

Plasma samples were collected from the heart at 8 weeks, between 7:00 am and 9:00 am, in tubes containing EDTA (TERUMO, Tokyo, Japan) [22]. Tubes were immediately placed on ice and then centrifuged at 2,000 × *g* for 10 min at 4°C. The supernatants were collected and stored at −80°C before use. Plasma corticosterone levels were determined using an EIA kit (Yanaihara Institute Inc., Shizuoka, Japan) according to the manufacturer’s instructions. The measurement was performed in duplicate, and both intra- and inter-assay coefficients of variation were <5%.

### Determination of Aβ40 and Aβ42 levels in the hippocampus

The left hippocampus was harvested, immediately frozen, and kept at −80°C. Approximately 150 mg of tissue was homogenized in 1 ml 70% formic acid [22,27]. Homogenates were centrifuged at 100,000 × *g* for 1 h to remove particulate material, and the supernatant was recovered and neutralized with 20-fold dilution in 1 M Tris base. Following neutralization, the Aβ40 and Aβ42 levels per total protein were determined using ELISA kits (Human/Rat β Amyloid (40) and Human/Rat β Amyloid (42) ELISA Kits, Wako Pure Chemical Industries, Ltd., Osaka, Japan) according to the manufacturer’s instructions [22]. The measurement was performed in duplicate, and both intra- and inter-assay coefficients of variation were <5%.

### Determination of gene expression with real-time reverse transcription-polymerase chain reaction (RT-PCR)

The right hippocampus was harvested, immediately frozen, and kept in RNAlater, an RNA stabilization solution (Ambion, Austin, TX) at −80°C until use for real-time RT-PCR [22]. Total RNA was isolated from the hippocampus samples using Trizol reagent (Invitrogen, Carlsbad, CA), according to the manufacturer’s instructions. The isolated RNA was quantified by measurement of the absorbance at 260 nm, and its purity was determined by the 260/280 nm absorbance ratio. Samples with a ratio >1.8 were used [24]. Total RNA (2 µg) was reverse transcribed with AMV Reverse Transcriptase (TAKARA, Shiga, Japan) at 42°C for 30 min. The prepared cDNA was diluted 10-fold with yeast RNA (10 µg/mL). Real-time PCR was performed using TOYOBO SYBR Green PCR Master Mix (TOYOBO, Osaka, Japan) and the Mx3000P Real-time QPCR System (Agilent Technologies, Tokyo, Japan) for 45 cycles at 95°C for 20 s, the appropriate annealing temperature for 20 s, and 72°C for 20 s. The primer sequences were glucocorticoid receptor (Gr), 5′-AGGCAGTGTGAAATTGTATCCCAC-3′ and 5′-GAGGCTTACAATCCTCATTCGTGT-3′ [28], and β-secretase (Bace1), 5′-GCATGATCATTGGTGGTATC-3′ and 5′-CCATCTTGAGATCTTGACCA-3′ [29]. The primers used to detect the internal control, β-actin, were 5′-TGTTGCCCTAGACTTCGAGCA-3′ and 5′-GGACCCAGGAAGGAAGGCT-3′ [30]. Expression of each gene is shown as the relative copy number ratio of the target gene to β-actin for each sample [22].

### Statistical analysis

All data analysis was done using a statistical software package (SPSS 15.0J for Windows; SPSS Japan, Tokyo, Japan). Comparisons between groups were made using the t test with Bonferroni correction. The level of significance was set at *P* < 0.0125. 

## Results

No significant differences were found in food consumption among the four groups of rats over the experimental period. Body weights (mean ± SD) for the C-WT, D-WT, C- ApoE(−/−), and D-ApoE(−/−) groups were 475.7 ± 53.1, 442.5 ± 55.0, 428.3 ± 30.6, and 430.0 ± 20.0 g at 8 weeks, respectively. These body weights were not significantly different among the groups.

No significant differences in the time to complete a task or the number of errors were found among the four groups (Figure 1). For both genotypes, significant differences in the reference memory between the C and D groups were observed at 8 weeks.

**Figure 2:**
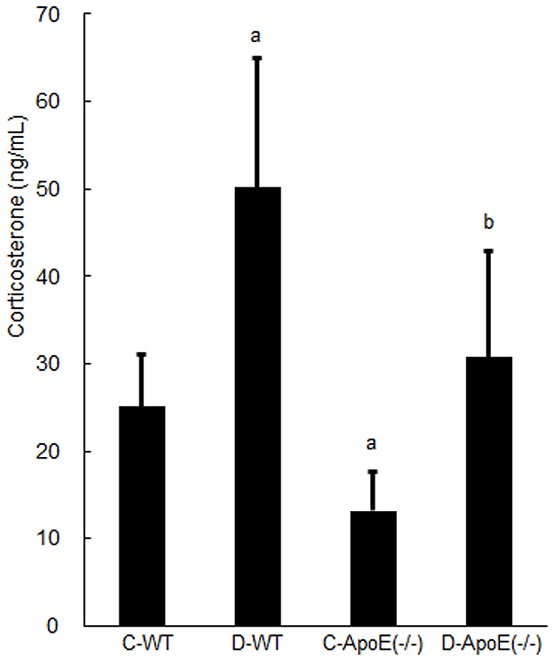
Serum levels of corticosterone in the four groups: C-WT, D-WT, C-ApoE(−/−), and D-ApoE(−/−). For each group, the results are expressed as the mean ± SD (n = 6). C, control; D, occlusal disharmony; WT, wild-type rats; ApoE(−/−), apoE-deficient rats. ^a^ *P* < 0.0125 compared with the C-WT group at 8 weeks (t test with Bonferroni correction). ^b^ *P* < 0.0125 compared with the C-ApoE(−/−) group at 8 weeks (t test with Bonferroni correction).

Plasma corticosterone levels in the two D groups were significantly higher than in the corresponding C groups (*P* < 0.0125) (Figure 2). The value in the C-WT group was significantly higher than that in the C-ApoE(−/−) group (*P* < 0.0125) (Figure 2).

**Figure 3:**
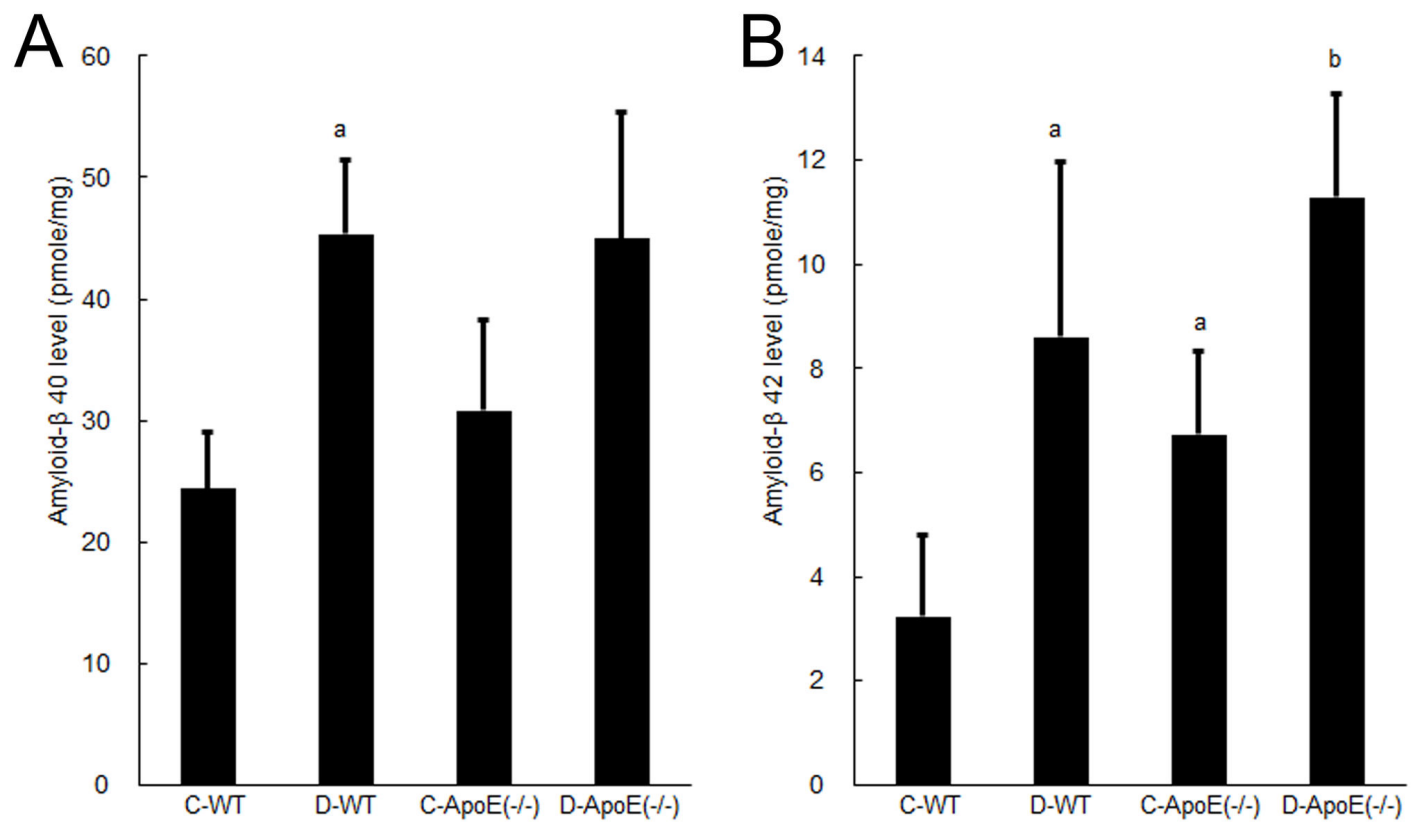
Amyloid-β40 (A) and 42 (B) levels in the hippocampus in the four groups: C-WT, D-WT, C-ApoE(−/−), and D-ApoE(−/−). For each group, the results are expressed as the mean ± SD (n = 6). C, control; D, occlusal disharmony; WT, wild-type rats; ApoE(−/−), apoE-deficient rats. ^a^ *P* < 0.0125 compared with the C-WT group at 8 weeks (t test with Bonferroni correction). ^b^ *P* < 0.0125 compared with the C-ApoE(−/−) group at 8 weeks (t test with Bonferroni correction).

The level of Aβ40 in the hippocampus was significantly higher in the D-WT group than that in the C-WT group (*P* < 0.0125) (Figure 3A). No significant differences were noted in the values between the C-ApoE(−/−) and D-ApoE(−/−) groups or between the two genotypes. In both genotypes, levels of Aβ42 in the hippocampus were significantly higher in the D groups than those in the C groups (*P* < 0.0125) (Figure 3B). The value in the C-ApoE(−/−) group was also significantly higher than that in the C-WT group (*P* < 0.0125), but we observed no significant difference between the D-WT and D-ApoE(−/−) groups (Figure 3B).

**Figure 4:**
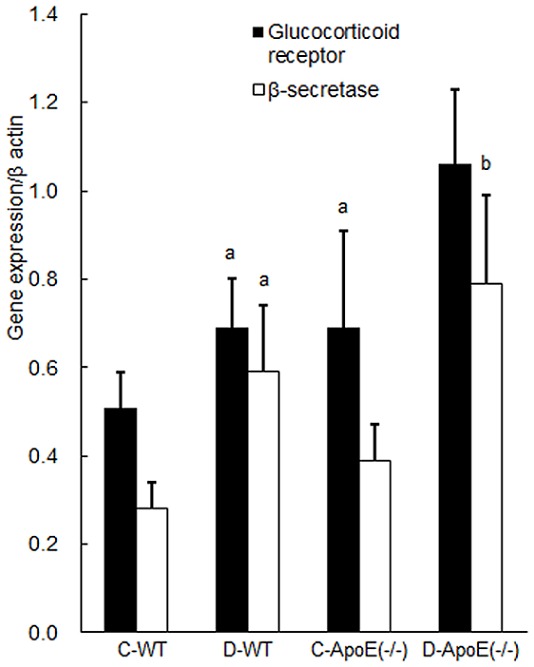
Gene expression/β-actin in the hippocampus in the four groups: C-WT, D-WT, C-ApoE(−/−), and D-ApoE(−/−). For each group, the results are expressed as the mean ± SD (n = 6). C, control; D, occlusal disharmony; WT, wild-type rats; ApoE(−/−), apoE-deficient rats. ^a^ *P* < 0.0125 compared with the C-WT group at 8 weeks (t test with Bonferroni correction). ^b^ *P* < 0.0125 compared with the C-ApoE(−/−) group at 8 weeks (t test with Bonferroni correction).

Gene expression of Bace1 was significantly higher in the D-WT and D-ApoE(−/−) groups than that in the C-WT and C-ApoE(−/−) groups, respectively (*P* < 0.0125) (Figure 4). On the other hand, we observed a significant difference in the Gr expression between the C-WT and D-WT groups and between the C-WT and C-ApoE(−/−) groups (*P* < 0.0125), and a similar trend was observed between the C-ApoE(−/−) and D-ApoE(−/−) groups (*P* = 0.022) (Figure 4).

## Discussion

LOAD is the most common cause of dementia in individuals [1]. The cause of this disease involves Aβ accumulation and its associated neurotoxicity [2]. However, little information is available about what upstream events cause the imbalance between Aβ production and clearance [5]. One of the strongest causes is inheritance of one or two ε4 alleles of the apoE gene [6]. ApoE4 is the major and most prevalent genetic risk factor for AD [6-10]. In addition to apoE, another risk factor for AD may be occlusal disharmony. In the rat model, psychological stress induced by occlusal disharmony reversibly induces Aβ accumulation in the rat hippocampus through glucocorticoid signaling [22]. However, the combined effects of apoE and occlusal disharmony on the pathways that regulate cellular levels of Aβ have not been analyzed. To the best of our knowledge, this is the first study to assess the combined effects of apoE deficiency and occlusal disharmony on Aβ accumulation in the rat hippocampus and on spatial memory.

Significantly increased levels of Aβ42 and Bace1 in the rat hippocampus were induced by occlusal disharmony in both WT and apoE-deficient rats. The reference memories in the D groups of both genotypes were significantly lower than in the corresponding C groups. In the D groups, we observed no significant differences in these values between WT and apoE-deficient rats. These results suggest that the effects of occlusal disharmony on Aβ accumulation and cognitive dysfunction were larger than those of apoE deficiency. In another mouse model, there is no significant difference in spatial learning between two genotypes (WT vs. apoE-deficient mice) under stressful experiences (repeated exposure to a rat) [31], a result that is similar to ours. Several investigators have attempted to explain the mechanism by which memory loss occurs under specific conditions of age, stress levels, and apoE status. The authors of some animal studies [31,32] have postulated that the effects of stress are mediated by glucocorticoids and depend on the presence of apoE. Although the effects of apoE may alter susceptibility to environmental factors such as stress [23], further studies are required investigate the mechanism of how occlusal disharmony appears to modulate the effects of apoE deficiency on dementia.

Our previous study showed that psychological stress induced by occlusal disharmony induces Aβ production in the rat hippocampus through glucocorticoid signaling [22]. In this study, we confirmed that occlusal disharmony induced Aβ production and Gr expression in the hippocampus and increased plasma corticosterone. Cognitive dysfunction was also induced by occlusal disharmony in our model. Previous reports have shown that the rat molar loss model causes memory impairment after tooth extraction [25,33]. On the other hand, a study using a mouse AD model reported that high levels of glucocorticoids found in AD mice play a central role in the development and progression of AD [34]. In that model, corticosterone elevates Aβ40 and Aβ42 levels through increases in Bace1. The primary mode of action by which glucocorticoids modulate Aβ levels appears to be mediated by binding to stress-activated Grs [34]. Aβ accumulation following occlusal disharmony may affect brain function through glucocorticoid signaling.

ApoE plays a dual role in Aβ clearance and deposition [20]. ApoE is involved in the clearance of Aβ from the central nervous system across the blood brain barrier [17,18]. On the other hand, amyloid precursor protein transgenic mice deficient in apoE show a dramatic decrease in fibrillary amyloid deposits, showing that apoE deficiency exerts a beneficial effect on Aβ fibrillogenesis and amyloid plaque formation in the mouse brain [35-40]. In this study, the level of Aβ42 in the C-ApoE(−/−) group was significantly higher than that in the C-WT group. This finding suggests that apoE deficiency alters Aβ clearance and then induces soluble Aβ accumulation, but not Aβ fibrillogenesis, in the rat hippocampus. Further studies are required to investigate the complex relationship between apoE deficiency and Aβ production, because whether increasing or decreasing human apoE levels is required to reduce Aβ levels remains unanswered [1].

No difference in spatial memory was noted between the C-WT and C-ApoE(−/−) groups in this study. Some studies show that apoE-deficient mice display a learning deficit in complex tasks involving hippocampal functions [41-46]. However, other groups have not detected cholinergic and behavioral deficits in apoE-deficient mice [47-50]. Another study showed that apoE-deficient mice perform better than controls in terms of reference memory and correct entries into the eight-arm maze [51]. Although inconsistent results regarding spatial memory in apoE-deficient mice have been reported, the reason may depend on the method of knockout, age, gender, or the stress system [52]

The level of plasma corticosterone in the C-WT group was significantly higher than that in the C-ApoE(−/−) group in our study, whereas the level of Gr in the C-WT group was significantly lower than that in the C-ApoE(−/−) group. A previous study suggested the hypothesis that genotypic differences in corticosterone secretion may be related to differences in negative feedback control, which are mediated by brain Gr [52]. The hypothesis is consistent with our results, although the details of the mechanism are still unknown [52].

Previous clinical studies suggest that tooth loss may affect dementia due to chronic rather than acute effects [53,54]. The present study showed increased levels of Aβ in the rat hippocampus and cognitive dysfunction due to occlusal disharmony over an 8-week period, which can be considered chronic effects. Although further studies are needed in humans, our current results suggest that tooth loss and/or molar loss could be related to initiation of dementia.

Our study has a limitation. We did not confirm the presence of amyloid plaques, and therefore, AD pathogenesis was not confirmed. Although apoE deficiency exerts a beneficial effect on Aβ fibrillogenesis and amyloid plaque formation in the mouse brain [35-40], the effects of occlusal disharmony on Aβ accumulation and cognitive dysfunction was larger than those of apoE deficiency, and there may be a possibility of amyloid plaques formation induced by occlusal disharmony. Therefore, a diagnosis of AD in these animal models will be required in long-term studies.

In conclusion, psychological stress induced by occlusal disharmony induced Aβ accumulation in the rat hippocampus and cognitive dysfunction through glucocorticoid signaling, and the effects of occlusal disharmony on Aβ accumulation and cognitive dysfunction were larger than those of apoE deficiency.
